# A review of clinical effects associated with metabolic syndrome and exercise in prostate cancer patients

**DOI:** 10.1038/pcan.2016.25

**Published:** 2016-06-28

**Authors:** J L Kiwata, T B Dorff, E T Schroeder, M E Gross, C M Dieli-Conwright

**Affiliations:** 1 Division of Biokinesiology and Physical Therapy, Ostrow School of Dentistry, University of Southern California, Los Angeles, CA, USA; 2 Norris Comprehensive Cancer Center, Keck School of Medicine, University of Southern California, Los Angeles, CA, USA; 3 Center for Applied Molecular Medicine, Keck School of Medicine, University of Southern California, Los Angeles, CA, USA

## Abstract

Androgen deprivation therapy (ADT), a primary treatment for locally advanced or metastatic prostate cancer, is associated with the adverse effects on numerous physiologic parameters, including alterations in cardiometabolic variables that overlap with components of the metabolic syndrome (MetS). As MetS is an established risk factor for cardiovascular mortality and treatment for prostate cancer has been associated with the development of MetS, interventions targeting cardiometabolic factors have been investigated in prostate cancer patients to attenuate the detrimental effects of ADT. Much support exists for exercise interventions in improving MetS variables in insulin-resistant adults, but less evidence is available in men with prostate cancer. Regular exercise, when performed at appropriate intensities and volumes, can elicit improvements in ADT-related adverse effects, including MetS, and contributes to the growing body of literature supporting the role of exercise in cancer survivorship. This review (1) discusses the biologic inter-relationship between prostate cancer, ADT and MetS, (2) evaluates the current literature in support of exercise in targeting MetS and (3) describes the physiological mechanisms by which exercise may favorably alter MetS risk factors in prostate cancer patients on ADT.

## Introduction

The 5-year relative survival for men with all stages of prostate cancer is 98.8% due in large part to recent therapeutic advances.^1^ With improved cancer-specific survival, consideration of treatment-related comorbidities has increased substantially. Androgen deprivation therapy (ADT) is one of the primary methods of treating prostate cancer. When used in combination with primary radiation for locally advanced or high-risk localized disease, ADT is associated with improved disease-free and overall survival.^2, 3^ However, due to the marked reduction in circulating testosterone, ADT is associated with detrimental changes to body composition, lipid profile and insulin sensitivity.^1^ Such components comprise the cardiometabolic condition known as metabolic syndrome (MetS). MetS is a group of cardiovascular risk factors including hypertension, central adiposity, hypertriglyceridemia, hyperglycemia and low high-density lipoprotein cholesterol (HDL-C) with insulin resistance (IR) serving as the underlying feature.^4^

MetS is an established risk factor for cardiovascular mortality, which contributes to 17.3 million deaths each year in the United States.^5^ It is present in >50% of men undergoing long-term ADT,^6^ potentially exposing this population to higher risk of the onset of cardiovascular disease (CVD) and mortality. Furthermore, MetS may contribute to prostate cancer behavior,^7^ suggesting a role for exercise not only in supportive care but potentially as part of cancer control therapy. Components of MetS are linked to an increased risk of prostate cancer and MetS-related biomarkers such as insulin, insulin-like growth factor-1 (IGF-1), leptin and adiponectin are implicated in the involvement of tumorigenesis.^7, 8^

Interventions targeting components of MetS would be expected to reduce the risk of cardiovascular comorbidity in cancer survivors and are particularly warranted to attenuate the detrimental cardiometabolic effects of ADT. One such intervention mode, exercise, is well supported in the literature for improving MetS variables in a variety of populations, including individuals with MetS, older adults and patients with type 2 diabetes mellitus.^9, 10, 11^ However, this area is largely understudied in men with prostate cancer, thus there is a lack of evidence to define the optimal use of exercise in prostate cancer patients receiving ADT. The purpose of this review is to (1) discuss the biologic inter-relationship between prostate cancer, ADT and MetS, and (2) describe the mechanistic implications for the utilization of exercise to target MetS and subsequent CVD risk.

## MetS and prostate cancer

MetS is a cluster of cardiovascular risk factors including hypertension, central adiposity, hypertriglyceridemia, hyperglycemia and low HDL-C with IR serving as the underlying feature. The definition of MetS by the International Diabetes Federation^4^ is shown in Table 1. With IR, normal levels of glucose are insufficient to produce a normal insulin response from fat, muscle and liver cells, often in the presence of central adiposity as a physical manifestation of this state. IR in fat cells causes hydrolysis of stored triglycerides and elevated levels of free fatty acids (FAs). These free FAs are absorbed by the liver resulting in increased triglycerides, low-density lipoprotein cholesterol, and decreased HDL-C. Furthermore, IR causes reduced glucose uptake in the muscle and decreased glucose storage in the liver, leading to the development of hyperglycemia.^12^

Various components of MetS including hyperinsulinemia,^13^ hypertension,^14^ central obesity^15^ and dyslipidemia^16^ have been linked to an increased risk of prostate cancer. Specifically, insulin, an integral component in a number of metabolic pathways such as lipogenesis, steriodogenesis and protein synthesis, elicits proliferative and anti-apoptotic actions in many cell types.^17, 18^ Insulin stimulates the production of IGF-1, a peptide growth factor, while suppressing the production of IGF-binding proteins (IGFBPs).^19^ As IGFBPs regulate the amount of circulating IGF-1 available to bind to the IGF-1 receptor, higher levels of insulin increase the amount of bioavailable IGF-1, provoking the signaling of growth or survival. In MetS, both hyperglycemia and hyperinsulemia increase bioavailable IGF-1 likely due to the suppression of IGBPs and increases in IGF-1 synthesis, respectively.^18^

Elevated circulating IGF-1 has been epidemiologically linked to risk of various cancer types including prostate cancer,^20, 21, 22^ and *in vivo* models implicate IGF-1 in prostate tumorigenesis.^7, 8, 23^ In addition, binding of IGF-1 to the IGF-1 receptor activates several pathways, such as the phosphatidylinositol 3-kinase (PI3K)/Akt and the Ras/mitogen-activated protein kinase pathways, that mitigate cell processes including cell growth, survival and angiogenesis (usually upregulated in 30–50% of prostate cancer) with downstream effects culminating in increased glucose metabolism, inhibition of apoptosis and stimulation of cell proliferation. The PI3K/Akt pathway is one of the most commonly altered pathways in epithelial cancers including prostate cancers.^24^ Of importance is the reciprocal feedback regulation in prostate cancer cells between androgen receptor signaling and signaling through the PI3K/Akt/mTOR pathway, which has been implicated in the emergence of androgen-independent tumors. Indeed, IGF-1 is able to stimulate androgen-sensitive and androgen-independent prostate cancer in human cell lines.^22^

MetS is associated with increased serum estradiol levels, decreased sex hormone-binding globulin concentration and decreased free testosterone levels. Higher concentrations of estrogen may promote testosterone-induced carcinogenesis and may be responsible for more aggressive prostate tumors.^25, 26^ MetS is associated with the production of various pro-inflammatory cytokines and growth factors, such as leptin and adiponectin, which may lead to genomic instability and a greater risk of cancer development.^27^ *In vitro*, leptin stimulates the growth of androgen-insensitive prostate cancer cells, whereas adiponectin inhibits angiogenesis. Thus, increased levels of leptin and lower levels of adiponectin contribute to the appearance of more advanced, less differentiated prostate tumors.^28^ Collectively, the aforementioned consequences of MetS on prostate cancer may pose detrimental effects on tumor development and severity, making MetS an important target for attenuation using interventions such as regular exercise.

## ADT and prostate cancer

Most patients with localized or regional prostate cancer treated at early stages have favorable outcomes, with a 5-year specific survival rate near 100%.^1^ Even patients with metastatic prostate cancer anticipate a median survival of 4 years, which has recently increased due to the improved treatment regimens. ADT, which may be attained through gonadotropin releasing hormone analogs (medical castration) or bilateral orchiectomy (surgical castration), is a cornerstone in the treatment of prostate cancer not only for men with metastatic disease but also as an adjunct to curative-intent therapy, particularly in high-risk disease.^29, 30, 31, 32^ Benefits of ADT are substantial and include objective tumor regression, extended survival, and relief of urinary symptoms and bone pain.^33, 34, 35^

However, a life-threatening health concern related to ADT use is its association with CVD and sudden cardiac death. Although confounded by differing population characteristics, some large observational studies have identified an association between ADT use and increased risk of CVD, myocardial infarction and sudden cardiac death.^36, 37, 38^ Thus, consideration of cardiovascular risk factors such as MetS is necessary due to the impact it may have on the development of CVD. The following section will focus on the effect of ADT on MetS as it pertains to cardiometabolic risk in men with prostate cancer on ADT.

## MetS and ADT

Specific components of MetS resulting from ADT that influence the development of diabetes and CVD include body composition, lipid profiles and IR.

### Body composition

Men with prostate cancer treated with ADT experience symptoms of hypogonadism with the adverse effects including metabolic alterations and unfavorable changes in body composition such as weight gain, loss of muscle mass, increased fat mass and decreased muscle strength.^39^ Because of the suppression of androgen synthesis and signaling, a reduction in lean mass and increase in fat mass, known as sarcopenic obesity, occurs as early as 3–6 months after initiation of ADT.^40, 41, 42^ A review of 16 studies reported an average 7.7% increase in body fat and 2.8% reduction in lean mass in men with prostate cancer on ADT, with longer duration of treatment associated with greater changes in body composition.^41^ Decreased lean mass is associated with reduced muscle strength and impaired physical performance.^43^

### Lipid profiles

Alterations in lipid profiles are highly variable during ADT use; however, many studies have reported increases in total cholesterol, triglycerides and HDL-C.^40, 44, 45^ The increase in HDL-C observed while on ADT is a beneficial consequence, although it is unknown whether this improvement provides a cardio-protective effect. Regardless, increases in HDL-C are seen as early as 3–6 months within initiation of treatment.^44, 45^ Because of the variability in lipid profile changes following ADT use, further investigation is warranted to determine the overall impact of ADT on cardiometabolic risk in this population.

### Insulin resistance

IR accompanies prediabetes, diabetes and obesity, and is an independent risk factor for CVD. ADT is associated with increased IR, independent of body composition and age.^46^ Compromised insulin sensitivity as a result of ADT occurs as an early consequence, increases in severity over time, and eventually culminates in hyperglycemia and finally type 2 diabetes.^37^ ADT further exacerbates insulin metabolism by increasing fasting insulin levels^44, 47^ and decreasing insulin sensitivity.^48, 49^ Changes in insulin sensitivity have been noted with short (12 weeks)^49^ or long-term (over 45 months)^46^ ADT use. Likely to exacerbate the observed development of IR are unfavorable changes in body composition, mainly increased visceral adiposity, following ADT use, thus resulting in a cardiometabolic adverse side effect such as MetS. In particular, the adipocytokines leptin and adiponectin, both secreted by adipose tissue, function to modulate insulin sensitivity and are elevated during ADT use.^48, 50^ Leptin is involved in appetite and the regulation of energy homeostasis, with levels secreted from adipose tissue in proportion to central adiposity. Circulating levels of leptin increase during ADT use, with or in the absence of increased fat deposition.^50^ The actions of adiponectin generally oppose those of leptin such that reductions in adiponectin levels are observed with obesity, type 2 diabetes, CVD, hypertension and MetS. Adiponectin is normally suppressed by testosterone,^51^ thus the observed increase in adiponectin during ADT is due to the lack of suppression by testosterone. Notably, the effects of adiponectin on insulin sensitivity occurring with ADT use does not overcome the effects of androgen deprivation on the prevalence of hyperinsulinemia.^48, 50^

### Managing cardiometabolic risk and MetS

CVD and diabetes are the leading causes of non-cancer deaths in prostate cancer survivors,^52^ thus it is imperative that men using ADT undergo assessments of their cardiometabolic profile before and intermittently during therapy to prevent MetS and additional comorbidities. The American Heart Association, American Cancer Society and American Urological Association have issued guidelines for men receiving ADT, which includes an evaluation of blood pressure, lipids and glucose within 3–6 months of initiating therapy with assessment annually for long-term therapy.^53^ Additional recommendations have been proposed, although not yet adopted, include: (1) maintenance of ideal body weight and waist circumference; (2) smoking cessation; (3) blood pressure control; (4) lipid control; (5) annual screening for diabetes mellitus; and (6) aspirin therapy with established CVD.^54^ Importantly, these recommendations may be mitigated through regular participation in exercise, thus the remainder of this review will focus on implications of exercise to attenuate MetS in prostate cancer survivors on ADT.

## Exercise as an intervention for MetS

Given the plausible association between prostate cancer, ADT and metabolic dysregulation, interventions targeting components of MetS may reduce the risk of cardiovascular comorbidity in cancer survivors as well as slow disease progression.^55^ Indeed, much support for improving MetS variables through exercise has been demonstrated in a variety of populations, including healthy adults, individuals with MetS, older adults and patients with type 2 diabetes mellitus.^9, 10, 11^ Yet, it is important to note that other lifestyle factors, including diet, smoking and alcohol use, may also contribute to MetS. As such, investigations have utilized a combination of exercise, diet and behavior counseling in their interventions, with the result of reducing MetS prevalence.^11^ As this review focuses on exercise as an intervention for MetS in prostate cancer survivors, the following sections will focus solely on the effects of exercise—rather than multiple lifestyle modifications—in improving MetS, the mechanisms underlying exercise-induced adaptations and the application of evidence-based exercise to survivorship care for patients on ADT.

### Exercise types

Of the exercise interventions, aerobic exercise possesses the greatest amount of support in improving cardiometabolic outcomes in individuals with MetS, albeit none were conducted in cancer survivors.^56, 57, 58, 59, 60^ Only two of six studies reported significant reductions in MetS prevalence, whereas all six reported improvements in individual MetS components.^56, 57, 58, 59, 60^ The HEalth, RIsk factors, exercise Training And GEnetics (HERITAGE) Family Study^60^ investigated the efficacy of 20 weeks of supervised aerobic training (AT) consisting of 3 days per week at 55% maximal oxygen uptake (VO_2max_) for 30 min and progressing to 75% VO_2max_ for 50 min. In their sample of 621 participants, 16.9% (105/621) met the ATP III criteria for MetS at baseline, and following the intervention, 30.5% (32/105) were no longer classified as having MetS. Similarly, the Studies of a Targeted Risk Reduction Intervention through Defined Exercise (STRRIDE) reported significant reductions in MetS prevalence in the exercise groups compared with a control group,^59^ with a low amount of moderate intensity AT (~19 km per week walking) or a high amount of vigorous intensity AT (~32 km per week jogging) improving MetS. In terms of individual MetS components, a reduction in waist circumference appears to be the most common improvement elicited by AT,^56, 57, 58, 59, 60^ whereas decreases in blood pressure,^57, 58, 59, 60^ decreases in triglycerides^59, 60, 61^ and increases in HDL-C level^56, 58, 59, 60^ are observed to a lesser extent.

Resistance exercise training (RT) has been utilized in MetS interventions, but primarily in combination with or compared with AT. A combined intervention of AT+RT appears to be at least as effective as AT alone in improving select MetS criteria.^56, 61^ However, in a sample of 86 adults, 8 months of RT, when compared with AT or AT+RT of the same duration, did not change the MetS *z-*score (a continuous score of the five MetS variables), whereas the MetS score was significantly reduced by AT and AT+RT.^61^

High-intensity interval training (HIT) has recently emerged as an effective approach to improving MetS criteria,^57, 62, 63^ and in one investigation,^57^ was superior to AT in eliciting improvements. HIT is characterized by bouts of short, vigorous activity interspersed by rest or intervals of low-intensity activity.^64^ Although vigorous activity could consist of either aerobic or resistance exercise, aerobic exercise has traditionally been the modality of choice in clinical populations. In a sample of 32 MetS patients, Tjonna *et al.*^57^ utilized four 4-min intervals of treadmill running at 90% heart rate max separated by 3-min active recovery intervals at 70% heart rate max and observed the removal of four out of five MetS risk factors. In comparison, patients who performed continuous aerobic exercise matched for the same training volume demonstrated improvements in only two MetS variables. In a different study using the same protocol, HIT was found to be no more effective at improving MetS criteria than RT or a HIT+RT protocol in a sample of 43 middle-aged adults with MetS.^62^ These findings suggest that HIT may be a promising alternative to commonly employed AT interventions, with the distinct advantages of having greater time efficiency and being more versatile, as either RT or AT may serve as the vigorous activity of choice. However, future work is needed to address the efficacy of RT, particularly in relation to HIT, in eliciting improvements in MetS variables.

## Exercise in prostate cancer patients on ADT

Although strong evidence exists for the efficacy of exercise training on cardiometabolic risk factors in individuals with MetS, there is less support for the impact of exercise on MetS outcomes in prostate cancer survivors. Numerous randomized controlled trials (RCTs) and uncontrolled trials (no randomization or control group) have been conducted on the benefits of exercise during ADT, although primary end points are commonly body composition or physical performance. The few RCTs that have examined the effect of exercise on cardiometabolic outcomes have generally focused on a single risk factor rather than the constellation of factors comprising MetS. Among those studies expressly targeting MetS criteria in prostate cancer survivors, none have reported reduced prevalence of MetS as a result of exercise. In total, eight RCTs examining at least one MetS component in prostate cancer survivors on ADT were found (Table 2) using an electronic search on peer-reviewed articles published between January 1990 and March 2016.^65, 66, 67, 68, 69, 70, 71, 72^ Studies were included if the trial was randomized and controlled, participants were prostate cancer patients on ADT, an exercise only intervention was performed rather than a combined intervention of exercise and diet or a self-report of physical activity behavior, and at least one MetS component served as a study end point.

Although the exercise modalities utilized by the eight studies have included AT+RT, AT, RT and HIT, only programs of AT+RT and HIT yielded changes in MetS variables.^67, 68, 70^ Culos-Reed *et al.*^68^ reported a significant difference in waist circumference between exercise and control groups following 16 weeks of an AT+RT intervention. Significant decreases in blood pressure were also observed, but only as a within-group difference in the exercise group following the intervention. Similarly, Cormie *et al.*^67^ observed a significant within-group increase in HDL-C in the exercise group after 12 weeks of combined AT+RT, but with no changes in blood pressure, fasting glucose or triglyceride levels. No significant between-group differences were observed for any variable when the AT+RT group was compared with the control group. Notably, in the first HIT intervention conducted in prostate cancer survivors on ADT, reductions in fasting glucose levels and increases in HDL-C were observed following 12 weeks of training; however, no between-group differences in MetS components were found between the exercise group and healthy control subjects.^70^

As nearly all eight interventions met the American Cancer Society's physical activity guidelines of at least 150 min per week of moderate intensity physical activity or at least 75 min per week of vigorous intensity physical activity,^73^ other factors, such as a higher weekly training volume or greater intensity of exercise, likely contributed to the greater efficacy of the above-mentioned exercise interventions. Culos-Reed *et al.*^68^ employed up to 5 days per week of moderate intensity home-based AT+RT, whereas Cormie *et al.*^67^ utilized a supervised program of progressive RT and moderate-to-vigorous intensity AT 2 days per week. Conversely, Hvid *et al.*^70^ used supervised AT, but employed HIT for 35 min each session, 3 days per week, at intensities ranging between 50 and 100% maximal oxygen uptake (VO_2max_). Therefore, it appears that home-based exercise may be as effective as supervised exercise in altering cardiometabolic variables in prostate cancer survivors provided training volume is high (that is, >150 min per week). In addition, shorter duration aerobic exercise can be as effective as longer duration aerobic exercise provided intensity is high (that is, 100% VO_2max_). Finally, it is important to note that factors relating to patient status, such as ADT duration, comorbid CVD or pharmacotherapy (that is, metformin), could potentially alter the efficacy of exercise interventions on cardiometabolic variables. Thus, to ascertain the optimal exercise prescription, future studies should adjust for these intervening factors through stratification or eligibility criteria.

Our laboratory is currently investigating the effects of a 12-week progressive RT intervention in prostate cancer patients on ADT (NCT01909440).^74^ In this RCT, the main inclusion criteria are serum testosterone concentration <50 ng dl^−1^ for study duration due to current or previous ADT, completion of radiation therapy >4 weeks before study enrollment, and recovery from major surgery within 6 months. Study end points are comprised of alterations in cardiometabolic variables including all MetS risk factors, body composition, physical function and quality of life. Participants randomized to the exercise group complete three sessions per week of supervised, whole-body RT using a model of systematic progression previously unemployed in exercise interventions for prostate cancer patients on ADT. The progression model, known as periodization, has been used primarily in athletic populations and is regarded as a superior method for optimally eliciting physiological and performance-related improvements.^75^ Periodized RT may serve as an ideal strategy for concomitantly increasing lean body mass while decreasing the fat mass, results not demonstrated with an AT-only intervention, thus optimizing beneficial changes in cardiometabolic variables. For this reason, RT, rather than AT, is utilized in our trial. The attention-control group receives a home-based stretching program and is offered the intervention following study completion. Findings from this study may provide an evidence-based exercise prescription for improving ADT-related adverse effects, including MetS, and contribute to the growing body of literature supporting the role of exercise in cancer survivorship. We are aware of other prospective studies involving exercise and prostate cancer survivorship with several studies incorporating novel ways to deliver exercise prescription, including recreational football-based training (NCT02430792),^76^ Xbox Kinect-based training (NCT01762241),^77^ supervised group AT+RT training (NCT02631681),^78^ or home-based training (NCT02248350).^79^

## Potential mechanisms of exercise-induced cardiometabolic improvements in prostate cancer patients on ADT

The relationship between MetS and prostate cancer centers on the role of insulin, IGF-1 and their receptors as key elements in downstream signaling pathways that promote tumor growth.^21^ Previous reviews on the molecular aspects of exercise-induced cardiometabolic improvements have focused on IR,^9, 11, 80, 81^ with exercise adaptations working to improve insulin sensitivity through the following mechanisms: increasing glucose tolerance,^9, 11^ increasing lipid oxidation,^9, 81^ and improving immune, metabolic and hormonal factors.^80, 82^ This section will briefly review the general mechanisms by which exercise may improve insulin action and favorably alter MetS risk factors in prostate cancer survivors on ADT.

### Glucose tolerance

Most support for exercise-induced improvements in insulin sensitivity arises from mechanistic studies in skeletal muscle investigating insulin signaling in response to AT. Enhanced glucose uptake in the muscle after exercise appears to be mediated by post-receptor insulin signaling and driven by glycogen repletion.^9, 11^ One signaling protein that has been studied in detail in response to exercise is glucose transporter isoform 4 (GLUT4), the major insulin-responsive glucose transporter that is highly expressed in the muscle as well as adipose tissue.^11^ In individuals with impaired glucose tolerance, AT has been found to increase GLUT4 protein concentration,^83, 84^ as well as lower blood glucose levels,^84^ increase glycogen synthase activity^83^ and increase glucose storage.^83, 84^ However, the activity of signaling proteins upstream of GLUT4 was not observed to increase after exercise training in both insulin-resistant and type 2 diabetic men.^83^ Furthermore, HIT has been shown to substantially increase GLUT4 protein content (~369%) and lower blood glucose levels in type 2 diabetes patients, even with a weekly training volume lower than the American Cancer Society recommendation.^85^ Studies investigating the mechanisms of skeletal muscle adaptation to HIT training have centered on the influence of peroxisome-proliferator activated receptor γ coactivator (PGC-1α), regarded as a major regulator of exercise-induced mitochondrial biogenesis and substrate utilization.^86^ Elevated expression of PGC-1α in the skeletal muscle following exercise has been shown to increase glucose uptake, GLUT4 expression and glycogen stores in a transgenic mouse model, whereas PGC-1α knockout mice exhibit delayed replenishment of glycogen stores after exercise.^86^ Although data from a meta-analysis of six prospective cohorts (*n*=274 125) did not identify a significant association between risk of fatal prostate cancer and impaired glucose tolerance,^87^ men with type 2 diabetes are more likely to present with high-grade prostate tumors,^88^ and exercise is an established method for reducing risk of type 2 diabetes.

### Lipid oxidation

Strong evidence exists for the role of consistent exercise in improving blood lipid profile through decreases in triglyceride and low-density lipoprotein levels, and increases in HDL levels.^89^ As IR has been linked to imbalances in lipid accumulation and oxidation in the skeletal muscle, exercise that alters this mismatch through changes in FA uptake or FA oxidation can significantly improve insulin sensitivity.^89^ These improvements are primarily mediated through changes in muscle substrate utilization that increase FA oxidation at rest and during submaximal exercise.^89^ An increased reliance on FA oxidation was observed at rest^90^ and during low-intensity aerobic exercise in insulin-resistant men after exercise training.^91^ Furthermore, oxidative enzymes responsible for FA oxidation were increased in obese middle-aged adults after a 4-month training program, and were accompanied by increases in insulin sensitivity.^92^ However, some authors have suggested that the reduced availability of plasma free FA, rather than FA uptake, may be responsible for improvements in insulin sensitivity.^90^ The availability of free FA may also be important in the context of prostate cancer as observational and preclinical data support FA availability to tumors leads to prostate cancer growth.^93, 94^ This is especially relevant in the setting of MetS, as altered blood lipid profiles increase FA availability, potentially fueling prostate tumors with energetic or biosynthetic substrates.^95^ Although the precise impact of exercise-induced increases in lipid oxidation on prostate cancer progression is not yet known, one may speculate that improvements in IR via reduced availability of FA may limit the ability of cancer cells to utilize exogenous free FA in their metabolic requirements.

### Immunometabolism

Dysregulation of the complex interaction between immune and metabolic factors due to excessive adiposity has been implicated in the pathogenesis of several chronic diseases, including type 2 diabetes, CVD and prostate cancer.^96, 97^ In particular, excess adipose tissue leads to the elevated production of pro-inflammatory adipokines, including leptin, tumor necrosis factor and interleukin-6 (IL-6), contributing to a persistent state of low-grade inflammation.^96^ Regular exercise has been proposed to counter this systemic inflammation through reductions in visceral fat and the subsequent decline in pro-inflammatory adipokines, as well as through the transient rise in anti-inflammatory myokines, or cytokines that are released from contracting the skeletal muscle during exercise.^80^ IL-6 has been identified as the primary myokine mediating the immunological and metabolic response to exercise, despite its pathogenic role in the development of MetS and other chronic disease.^98^ In particular, although high resting plasma levels of IL-6 are associated with MetS, AT has been shown to decrease resting plasma IL-6 levels and increase expression of its receptor, IL-6rα, such that sensitivity to IL-6 is increased in the skeletal muscle.^99^ These observations have led one group of authors to hypothesize that physical inactivity leads to elevated plasma levels of IL-6 and therefore IL-6 resistance, which parallels elevated plasma levels of insulin and IR.^82^ This hypothesis is supported by evidence demonstrating the beneficial effect of IL-6 on insulin sensitivity. Specifically, infusion of IL-6 has been shown to increase glucose uptake and translocation of GLUT4 in rat L6 muscle myotubes^100^ and enhance whole-body insulin sensitivity during a hyperinsulinemic-euglycemic clamp in healthy humans.^100^ In addition, elevations in the anti-inflammatory cytokine IL-10 following exercise, attributed to contraction-mediated rises in IL-6, are purported to increase insulin sensitivity,^80^ as treatment with IL-10 was demonstrated to attenuate IR following acute lipid infusion in mice.^101^ Collectively, these findings suggest that IL-6 released from contracting muscle modulates positive metabolic and immune responses that work to improve IR, and possibly MetS. The dynamic changes in IL-6 due to exercise may also serve a beneficial role in tumorgenesis, as consistent aerobic exercise has been shown to decrease tumor growth through intratumoral infiltration by IL-6-sensitive natural killer cells.^102^ Furthermore, aerobic exercise favorably altered metastatic gene expression while decreasing resting levels of IL-6 in an orthotopic model of murine prostate cancer, suggesting reductions in resting levels of IL-6 may be related to tumor regression to a less aggressive phenotype.^103^ Certainly, further research is needed to elucidate exercise-induced changes in inflammation and its relation to prostate cancer progression.

### IGF-1:IGFBP

Epidemiological studies have demonstrated a significant association between high IGF-1 levels, low IGFBP levels and increased risk of prostate cancer,^21^ spurring investigations into the effects of exercise on prostate tumor growth and cancer progression. Both insulin and IGF-1 have been shown to suppress the production of sex hormone-binding globulin, resulting in increased levels of free testosterone that may stimulate prostate tumor cell growth.^104^ Several investigations have provided support for alteration of IGF axis factors through acute^105^ and chronic exercise training.^106, 107, 108, 109^ In particular, serum from healthy men who performed a single bout of exercise^105^ or regular aerobic exercise for at least 5 days^106, 107, 108, 109^ was found to reduce proliferation and increase apoptosis of serum-stimulated lymph node cancer of the prostate tumor cells *in vitro.* Furthermore, the exercise training reduced serum insulin and IGF-1 levels, and increased IGFBP-1 and sex hormone-binding globulin levels. When IGFBP-1 was added back to the exercise serum, lymph node cancer of the prostate growth was reduced and apoptosis was increased.^109^ Although serum in these aforementioned experiments was obtained from healthy males, a recent study expanded these methods to prostate cancer.^71^ A sample of 13 prostate cancer survivors on ADT performed 6 months of RT, which was found to significantly increase serum IGFBP-3 and reduce IGF-1:IGFBP-3 ratio in comparison with a control group. Although it appears that chronic RT beneficially alters factors in the IGF axis to reduce bioavailable IGF-1, larger RCTs are needed to ascertain the clinical impact of these results on the progression of prostate cancer.

## Conclusion

**Figure 1:**
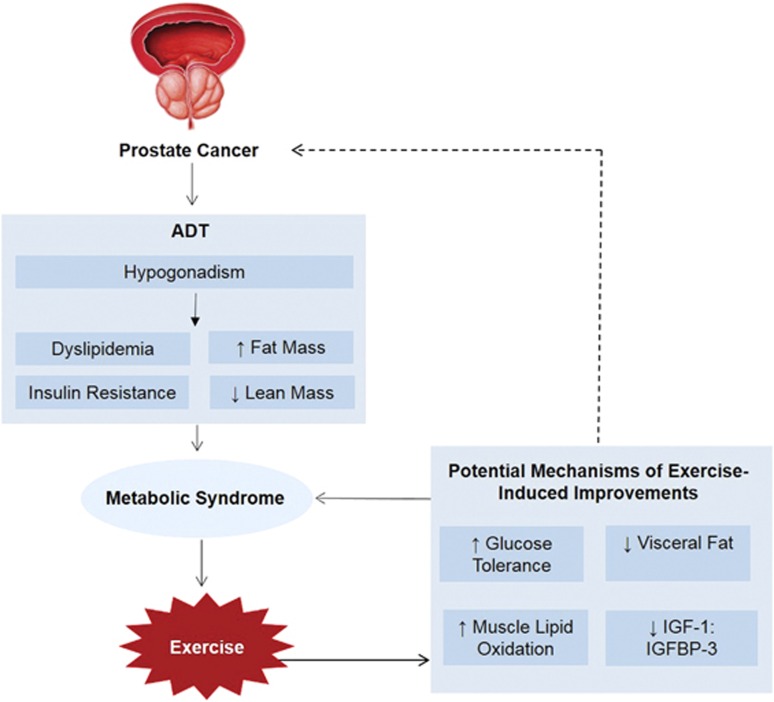
Relationship between prostate cancer, androgen deprivation therapy (ADT), metabolic syndrome and potential mechanisms of improvement due to exercise. IGF, insulin-like growth factor; IGFBP, IGF-binding protein.

Currently, there is insufficient evidence to directly indicate specific exercise guidelines to ameliorate MetS risk factors in prostate cancer patients on ADT. As ADT results in severe hypogonadism, and hypogonadism is an independent risk factor for MetS, ADT-induced metabolic complications are a significant issue in survivorship care (Figure 1). Furthermore, MetS is associated with worse prognosis in prostate cancer patients, including higher tumor grade, recurrence and increased mortality.^55^ Yet, a substantial body of work exists in support of exercise as a therapeutic intervention for favorably altering MetS variables, albeit in populations other than cancer survivors. In addition, exercise interventions conducted in prostate cancer survivors on ADT suggest that various forms of exercise programs, including resistance, aerobic and high-intensity interval training, are safe and well tolerated by patients, and may beneficially modify cardiometabolic risk factors. Thus, the features of interventions that elicit positive changes in MetS variables can be summarized to outline a possible exercise prescription (Table 3). Although large-scale RCTs are needed to ascertain the precise exercise prescription necessary to improve MetS factors arising from ADT administration, patients can still benefit from consistent exercise as improvements in muscle mass, strength, physical function and psychological well-being have been demonstrated.^68, 69, 72^ Therefore, participation in an appropriately designed exercise program may confer additional health benefits and should be encouraged for prostate cancer survivors receiving ADT.

## Figures and Tables

**Table 1:** The International Diabetes Federation Definition of Metabolic Syndrome

*Central obesity (defined as waist circumference with ethnicity-specific values) plus any two of the following four factors:*	
Waist circumference (white males)	⩾94 cm
Elevated fasting plasma glucose	⩾100 mg dl^−1^, or previously diagnosed type 2 diabetes
Reduced HDL cholesterol	<40 mg dl^−1^, or specific treatment for this lipid abnormality
Elevated blood pressure	Systolic⩾130 or diastolic⩾85 mm Hg, or treatment of previously diagnosed hypertension
Elevated triglycerides	⩾150 mg dl^−1^, or specific treatment for this lipid abnormality

Abbreviation: HDL, high-density lipoprotein.

**Table 2:** Summary of exercise RCTs evaluating MetS in prostate cancer patients on ADT

*Study*	*Year*	*Sample*	*Age (years) (mean±s.d.)*	*Treatment*	*Study duration*	*Exercise intervention*	*Frequency (days per week)*	*Key MetS findings*
Bourke *et al.*^66^	2014	Exe (*n*=50) Con (*n*=50)	Exe (71.0±6.0) Con (71.0±8.0)	ADT ⩾6 months and expected to receive ADT for study duration	12 weeks	Small-group supervision of PRT and PAT including lifestyle advice seminars PRT: 2–4 sets of exercises at 8-12 reps starting at 60% RM PAT: 30 min at 55–75% HRmax and RPE 11–13	3	Exe vs control BP ↔
Bourke *et al.*^65^	2011	Exe (*n*=25) Con (*n*=25)	Exe (71.3±6.4) Con (72.2±7.7)	ADT ⩾6 months	12 weeks	Home-based and group supervision of RT and AT including dietary advice seminars RT: 2–4 sets of exercises incorporating large muscle groups at light-to-moderate intensity AT: 30 min at 55–85% HRmax and RPE 11–15	Up to 5 encouraged	Exe vs control BP, waist to hip ↔
Cormie *et al.*^67^	2015	Exe (*n*=32) Con (*n*=31)	Exe (69.6±6.5) Con (67.1±7.5)	No prior ADT and expected to receive ADT for 3 months	12 weeks	Small-group supervision of PRT and PAT PRT: 1–4 sets of 8 exercises at 6–12 RM incorporating major muscle groups PAT: 20–30 min at 70–85% HRmax	2	Exe vs control BP, glucose, HDL, TG ↔ Pre-to-post Exe HDL ↑ BP, glucose, TG ↔
Culos-Reed *et al.*^68^	2010	Exe (*n*=53) Con (*n*=47)	Exe (67.2±8.8) Con (68.0±8.4)	Expected to receive ADT for at least 6 months	16 weeks	Home-based and weekly group sessions consisting of walking, stretching and Theraband exercises at a moderate intensity	3–5 encouraged	Exe vs control BP ↔ WC ↓ Pre-to-post Exe BP ↓ WC ↔
Galvao *et al.*^69^	2010	Exe (*n*=29) Con (*n*=28)	Exe (69.5±7.3) Con (70.1±7.3)	ADT ≥ 2 months and expected to receive ADT for 6 months	12 weeks	Supervised PRT and PAT PRT: 2–4 sets of 8 exercises incorporating major muscle groups at 12–6 RM PAT: 15–20 min at 65–80% HRmax and RPE 11–13	2	Exe vs control Glucose, HDL, TG ↔
Hvid *et al.*^70^	2011	Exe (ADT; *n*=9) Con (healthy; *n*=10)	Exe (67.8±6.4) Con (68.5±3.5)	ADT ⩾3 months	12 weeks	Supervised HIT: 35 min interval training 65–100% VO_2max_	3	Exe vs control HDL, glucose, TG ↔ Pre-to-post Exe HDL ↑ Glucose↓ TG ↔
Segal *et al.*^72^	2003	Exe (*n*=82) Con (*n*=73)	Exe (68.2±7.9) Con (67.7±7.5)	ADT for study duration	12 weeks	Supervised PRT PRT: 2 sets of 9 exercises incorporating major muscle groups starting at 60–70% RM.	3	Exe vs control WC ↔
Santa Mina *et al.*^71^	2013	AT (*n*=13) RT (*n*=13)	AT (70.6±8.1) RT (73.6±8.8)	ADT for study duration	6 months	Home-based RT or AT RT: 10 exercises incorporating major muscle groups AT: 60 min at 60–80% HRmax	Up to 5 encouraged	AT vs RT WC ↔

Abbreviations: ADT, androgen deprivation therapy; AT, aerobic training; BP, blood pressure; Con, control; Exe, exercise; HDL, high-density lipoprotein; HIT, high-intensity interval training; HRmax, heart rate max; MetS, metabolic syndrome; PAT, progressive aerobic training; PRT, progressive resistance training; RCT, randomized controlled trial; RM, repetition maximum; RPE, rating of perceived exertion; RT, resistance training; TG, triglycerides; WC, waist circumference.

**Table 3:** Summary of evidence-based exercise recommendations for improving MetS variables in prostate cancer patients on ADT

	*Frequency (days per week)*	*Intensity*	*Duration*	*Description*
Aerobic+resistance training^67, 68^	Up to 5	Moderate to vigorous	60 min	Aerobic exercise 20–30 min of walking, rowing or cycling at 75–80% estimated HRmax Resistance exercise Exercises targeting major muscle groups performed for ~3 sets of 6–12 RM
High-intensity aerobic interval training^70^	3	55–100% VO_2max_	35 min	5–25-min intervals of cycle ergometry

Abbreviations: ADT, androgen deprivation therapy; HRmax, heart rate max; MetS, metabolic syndrome; RM, repetition maximum; VO_2max_, maximal oxygen uptake.
